# Morphological and molecular characterization of *Sarcocystis chenggongensis* n. sp. (Apicomplexa: Sarcocystidae) in domestic sheep (*Ovis aries*) from China

**DOI:** 10.1186/s13071-026-07289-1

**Published:** 2026-03-09

**Authors:** Junjie Hu, Luyao Qian, Danqu Lamu, Shuangsheng Deng, Yuning Zhang, Yurong Yang

**Affiliations:** 1 School of Ecology and Environmental Sciences and Yunnan Key Laboratory for Plateau Mountain Ecology and Restoration of Degraded Environments, Yunnan University, Kunming, 650091 China; 2 Institute of Animal Science, Tibet Academy of Agricultural and Animal Husbandry Sciences, Lhasa, 850009 China; 3 Joint Laboratory of Virology and Immunity, School of Biological Sciences, Yunnan University, Kunming, 650091 China; 4 College of Veterinary Medicine, Henan Agricultural University, Zhengzhou, 450046 China

**Keywords:** *Sarcocystis chenggongensis*, *Ovis aries*, Ultrastructure, *18S rDNA*, *28S rDNA*, *ITS-1*, *cox1*, *rpoB*

## Abstract

**Background:**

Infections with *Sarcocystis* spp. in sheep (*Ovis aries*) are globally prevalent and pose significant health and economic concerns. Nine *Sarcocystis* taxa, comprising seven identified species and two unclassified entities, are known to form sarcocysts in sheep. Macroscopic sarcocysts with elongated villar protrusions are rare, having been reported only twice: as *S. mihoensis* in Japan and as an *S. mihoensis* -like organism in Spain. This study reports the first identification of this morphological type in China.

**Methods:**

Muscle samples were collected from 83 domestic sheep in Kunming City, China, between March and September 2025. Sarcocysts were characterized morphologically using light microscopy (LM) and transmission electron microscopy (TEM). For molecular analysis, genomic DNA was extracted from individual sarcocysts isolated from different sheep. Five genetic markers—the nuclear *18S rDNA*, *28S rDNA*, and *ITS-1* regions, the mitochondrial *cox1* gene, and the apicoplast *rpoB* gene—were amplified, sequenced, and analyzed.

**Results:**

A novel sarcocyst type was detected via LM in 6 out of 83 (7.2%) domestic sheep. These macroscopic sarcocysts measured up to 6230 μm in length and 341 μm in width, and possessed a thick cyst wall with numerous sloping villar protrusions (VPs) measuring 6.9–11.9 μm in length. Ultrastructurally, the VPs were 5.8–9.3 μm long and 0.8–1.1 μm wide, lined by an electron-dense layer, and contained scattered microtubules that extended from the apex to the base. Sequence comparisons with GenBank entries revealed the highest sequence similarities with *S. buffalonis* for *18S rDNA* (97.6–98.0%), *S. miescheriana* for *28S rDNA* (92.1–92.3%), *S. japonica* for *cox1* (81.2–81.8% identity), and *S. arctica* for *rpoB* (88.5–88.8% identity). No significant matches were found for the *ITS-1* region. Genetic divergence analysis against other sheep-infecting species indicated the smallest distances with *S. medusiformis* at *18S rDNA* (0.0582), *S. gigantea* at *28S rDNA* (0.0556), and *S. gigantea* at *cox1* (0.2922). Given the distinct morphological features and the unique molecular characteristics, this organism is proposed as a new species, *Sarcocystis chenggongensis* n. sp.

**Conclusions:**

The discovery of this new species marks the third global report of macroscopic sarcocysts with elongated villar protrusions, confirming a broad geographical distribution for this rare morphotype. Persistent taxonomic uncertainties, due to inconsistent data, require future research to resolve the group’s evolution and life cycles.

**Graphical Abstract:**

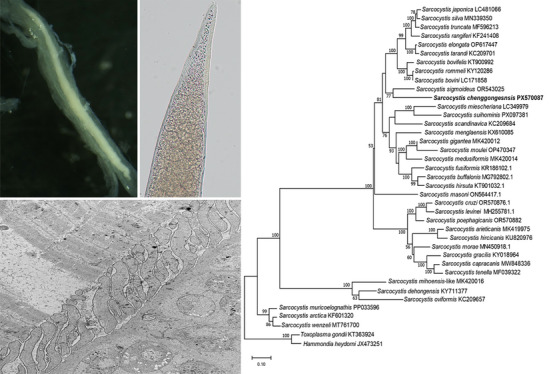

## Background

*Sarcocystis* spp. are cyst-forming, obligate intracellular protozoan parasites with a two-host life cycle involving a predator (definitive host) and its prey (intermediate host). Sexual reproduction occurs in the intestinal mucosa of definitive hosts, producing oocysts, while asexual replication in intermediate hosts results in sarcocyst formation in muscles and other tissues [1]. *Sarcocystis* infections in domestic sheep (*Ovis aries*) are globally prevalent and pose significant health and economic concerns. Clinical disease, particularly in lambs, can include encephalitis, myocarditis and myositis, manifesting as muscle weakness, anorexia, ataxia, tremors, and generalized weakness, sometimes with a fatal outcome [2–4]. Furthermore, entire carcasses or meat cuts from infected adult sheep may be condemned at slaughter due to macroscopic sarcocysts and associated eosinophilic myositis, resulting in substantial economic losses [5, 6].

To date, seven *Sarcocystis* species have been confirmed to form sarcocysts in domestic sheep: *S. tenella* Moulé, 1886 [7]; *S. arieticanis* Heydorn, 1985 [8]; *S. gigantea* Ashford, 1977 [9]; *S. medusiformis* Collins et al., 1979 [10]; *S. mihoensis* Saito et al., 1997 [11]; *S. microps* Wang et al., 1988 [12]; and *S. cystiformis* Wang et al., 1989 [13]. Additionally, two morphologically or molecularly distinct but unclassified species have been provisionally designated as *S. gracilis*-like [14] and *S. mihoensis*-like [15]. Among these, *S. tenella*, *S. arieticanis*, and *S. gigantea* are globally distributed, while *S. medusiformis* has been reported only in limited regions [1, 16]. In contrast, *S. mihoensis*, *S. mihoensis*-like, *S. gracilis*-like, *S. microps*, and *S. cystiformis* have each been documented in just a single study. Consequently, *S. tenella*, *S. arieticanis*, *S. gigantea*, and *S. medusiformis* have been well characterized morphologically and molecularly, with extensive nucleotide sequences available in public databases. However, except for *S. mihoensis*-like, no genetic sequences exist for the remaining species.

In China, three *Sarcocystis* species in sheep—*S. tenella*, *S. arieticanis*, and *S. gigantea*—have been unequivocally confirmed through both morphological and molecular analyses [17, 18]. In the 1980s, a study from Qinghai Province reported two additional species, *S. microps* and *S. cystiformis* [12, 13]; however, these were described with limited supporting data, and their existence has not been independently verified, leaving their taxonomic validity uncertain.

Given this unresolved taxonomy of *Sarcocystis* spp. in domestic sheep, we launched a long-term surveillance program to monitor infections and collect samples for genomic and cell culture studies. During this investigation, we identified a novel *Sarcocystis* species, characterized by distinct morphological and molecular traits.

## Methods

### Sample collection and sarcocyst isolation

Skeletal muscle samples (approximately 100 g each) were collected from 83 domestic sheep between March and September 2025 at local farmers’ markets in Chenggong District, Kunming City, Yunnan Province, China. Each sample represented a different animal. To screen for sarcocysts, ten muscle fragments (approximately 10 × 3 mm each) from each sample were compressed between glass slides and examined under a stereomicroscope (Olympus SZX16, Tokyo, Japan). Detected sarcocysts were carefully dissected from the muscle tissue using fine needles for subsequent morphological and molecular analysis.

### Morphological observation

Isolated sarcocysts were initially examined using an Olympus BX53 light microscope (Tokyo, Japan). For detailed histology, small muscle specimens (approximately 1 × 0.5 × 0.5 cm) containing sarcocysts were fixed in 10% buffered formalin, routinely processed, embedded in paraffin, sectioned at 4 µm, and stained with H&E. For TEM, samples from two animals containing a total of six sarcocysts were fixed in 2.5% buffered glutaraldehyde. These samples were then post-fixed in 1% osmium tetroxide, dehydrated through a graded ethanol series, and embedded in an Epon-Araldite mixture. Ultrathin sections were stained with uranyl acetate and lead citrate and examined using a JEM100-CX transmission electron microscope (JEOL Ltd., Tokyo, Japan) at 80 kV.

### DNA isolation, PCR amplification, and sequencing

Genomic DNA was extracted from four individual sarcocysts, each from a different sheep, using the TIANamp Genomic DNA Kit (Tiangen Biotech, Beijing, China) according to the manufacturer’s instructions. For precise species identification, five genetic loci were targeted for amplification: *18S rDNA*, *28S rDNA*, *ITS-1* region, and the mitochondrial *cox1* and apicoplast *rpoB* genes. Near-complete *18S rDNA* was amplified using primer pairs SarAF/SarBR and SarCF/SarDR [19], while near-full-length *28S rDNA* was amplified with primers KL1/KL3, KL4/KL5b, and KL6a/KL2 [20]. The complete *ITS-1* region was amplified with primers SU1F/5.8SR2 [21], and partial fragments of the *cox*1 and *rpoB* genes were amplified using SF1/SR4 [22] and RpoBF/RpoBR [23], respectively.

Polymerase chain reaction (PCR) amplifications were performed in a 25 µL reaction mixture containing 12.5 µL of 2 × Taq Plus Master Mix II (Zanna Biotech Ltd., Kunming, China), 1 µL of each primer (10 µM), 9.5 µL of ddH_2_O, and 1 µL of DNA template. The thermal cycling profile consisted of an initial denaturation at 95 °C for 5 min; followed by 35 cycles of denaturation at 95 °C for 30 s, annealing at 55 °C for 30 s, and extension at 72 °C for 1 min; with a final extension at 72 °C for 5 min. Amplified products were gel-purified, cloned, and four clones per gene from distinct sarcocysts were sequenced and assembled as previously described [24].

### Molecular analysis

Sequence identity among the newly obtained sequences was assessed using the Sequence Identity Matrix tool in BioEdit (Version 7.7.1). Initial GenBank screening and pairwise similarity comparisons of the sequences were conducted using the web-based Basic Local Alignment Search Tool (BLASTn) available on the National Center for Biotechnology Information (NCBI) website. Pairwise genetic distances between the newly generated sequences and those of previously reported *Sarcocystis* spp. from sheep in GenBank were computed in MEGA 12 [25] using the maximum composite likelihood model (Compute Pairwise Distances function).

### Phylogenetic analysis

Phylogenetic analyses of the *18S rDNA*, *28S rDNA*, and *cox1* gene were conducted separately in MEGA 12, utilizing *Sarcocystis* spp. sequences retrieved from the GenBank database. Multiple sequence alignments for each locus were generated using the ClustalW algorithm with the following parameters: for pairwise alignments, the gap opening penalty was set at 10 and the gap extension penalty at 10; for multiple alignments, the gap opening penalty was 0.1 and the gap extension penalty was 0.2. The resulting alignments were manually inspected and trimmed, yielding final datasets with lengths of 2082 base pairs (bp) for *18S rDNA*, which included 36 sequences representing 34 taxa; 3808 bp for *28S rDNA*, consisting of 24 sequences from 21 taxa; and 1046 bp for *cox1*, containing 39 sequences from 39 taxa.

Maximum likelihood (ML) trees were constructed for each gene. The best-fit nucleotide substitution models, determined by MEGA 12’s “Find Best DNA/Protein Models” program, were the Hasegawa-Kishino-Yano model for *18S* and *28S rDNA* and the General Time Reversible model for *cox1*. Gaps and missing data were handled by complete deletion. The robustness of the phylogenetic trees was evaluated through 1000 bootstrap replicates. All phylogenetic trees were rooted using *Hammondia* spp. and *Toxoplasma gondii* as outgroup taxa.

## Results

### LM and TEM observations on sarcocysts

**Figure 1:**
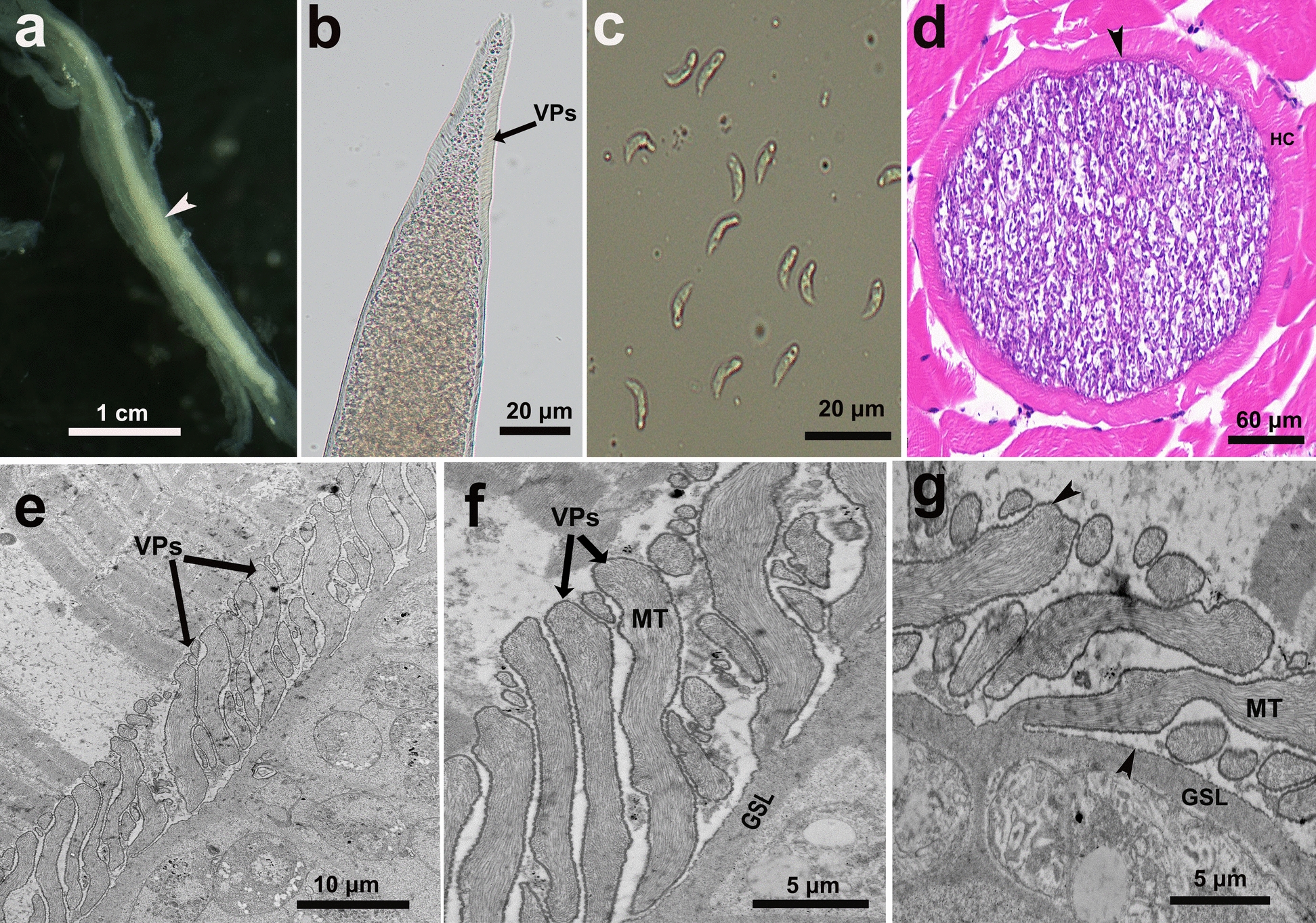
Morphology of *Sarcocystis chenggongensis* n. sp. sarcocysts from skeletal muscles of infected sheep, observed by LM (**a**–**d**) and TEM (**e**–**g**). **a** Sarcocyst (arrowhead) within skeletal muscle (unstained). **b** Isolated sarcocyst (unstained), showing sloping VPs on the cyst wall. **c** Banana-shaped bradyzoites released from a sarcocyst (unstained). **d** Cross-section of a sarcocyst (H&E staining), showing the sarcocyst surrounded by a HC, and the thick, striated cyst wall (arrowhead). **e** Longitudinal section of a sarcocyst, illustrating VPs on the cyst wall surface. **f** Longitudinal section showing scattered MT distributed along the full length of the VPs, from apex to base, but not extending into the GSL. **g** Diagonal section of a sarcocyst. Note the invaginations (arrowhead) presented on the surfaces of both the VPs and the GSL

Sarcocysts of the new species were detected in 6 of 83 (7.2%) examined domestic sheep. Under LM, the macroscopic sarcocysts were fusiform and tapered toward both ends, measuring 5760–6230 μm in length and 215–341 μm in width (*n* = 15) (Fig. 1a). The cyst wall was thick and exhibited numerous sloping VPs, which measured 6.9–11.9 μm in length (*n* = 30) (Fig. 1b, d). Internally, the sarcocysts were compartmentalized by septa and densely packed with banana-shaped bradyzoites measuring 11.2–13.7 μm × 1.6–2.5 μm (*n* = 20) (Fig. 1c).

Ultrastructurally, the sloping VPs measured 5.8–9.3 μm in length and 0.8–1.1 μm in width, and were lined by an electron-dense layer (Fig. 1e–g). Scattered microtubules were observed within the VPs, traversing their length from apex to base but without extending into the underlying GSL (Fig. 1f, g). The GSL was 0.7–1.0 μm thick. Invaginations presented on the surfaces of both the VPs and the GSL (Fig. 1f, g). Overall, the ultrastructure of the sarcocyst wall was consistent with the morphological classification of Type 10f, as defined by Dubey et al. (2016) [1].

### Molecular characterization

Sequences of the *18S rDNA*, *28S rDNA*, *ITS-1*, *cox1*, and *rpoB* genes were obtained from sarcocysts of the new species isolated from domestic sheep. The four *18S rDNA* sequences (1879–1883 bp) shared 99.5–100% identity (mean 99.7%), and the four *28S rDNA* sequences (3459–3480 bp) showed 98.2–99.9% identity (mean 98.8%). The four *ITS-1* sequences (898–905 bp) exhibited 97.1–98% identity (mean 97.5%), whereas *cox1* (1060 bp) and *rpoB* (511 bp) were identical (100%) among all isolates. These sequences have been deposited in GenBank under accession numbers: three *18S rDNA* (PX625798–PX625800), four *28S rDNA* (PX625794–PX625797), four *ITS-1* (PX632648–PX632651), one *cox1* (PX570087), and one *rpoB* (PX619186).

BLAST analysis of the newly generated sequences against the GenBank database identified the closest homologs for each locus. The *18S rDNA* sequences showed 97.6–98.0% identity with *S. buffalonis* from water buffalo (*Bubalus bubalis*), and the *28S rDNA* sequences shared 92.1–92.3% identity with *S. miescheriana* from domestic pigs (*Sus scrofa*). The *cox1* sequence was most similar (81.2–81.8% identity) to that of *S. japonica* from sika deer (*Cervus nippon*), and the *rpoB* sequence exhibited 88.5–88.8% identity with *S. arctica* from red foxes (*Vulpes vulpes*) (Table 1). No significant homologs were detected for the *ITS-1* region.

**Table 1:** Nucleotide sequences similarities between *Sarcocystis chenggongensis* n. sp. and close-related *Sarcocystis* species in GenBank

DNA regions	Accession numbers (length, bp)	Identity with nucleotide sequences deposited in GenBank		
		*Sarcocystis* sp. (accession numbers)	% Query coverage	% Identity (average)
*18S rDNA*	PX625798–PX625800 (1879–1883)	*S. buffalonis* (KU247901–KU247913, AF017121)	96	97.6–98.0 (97.8)
		*S. hirsuta* (AF017122, KT901156–KT901164)	96	97.5–97.8 (97.6)
*28S rDNA*	PX625794–PX625797 (3459–3480)	*S. miescheriana* (MK867456–MK867458)	100	92.1–92.3 (92.2)
		*S. suihominis* (MK867471–MK867473)	100	91.5–91.9 (91.6)
*cox1*	PX570087 (1060)	*S. japonica* (LC349943–LC349976, LC481036– LC481065)	88–90	81.2–81.8 (81.5)
		*S. rangiferi* (KF241383– KF241409, KC209671–KC209676)	88	81.2–81.5 (81.3)
*rpoB*	PX619186 (511)	*S. arctica* (MF596311–MF596331)	69	88.5–88.8 (88.7)

### Genetic divergence between the novel *Sarcocystis* species and other sheep-infecting* Sarcocystis* spp.

To assess the genetic divergence between the newly discovered *Sarcocystis* species and related sheep-infecting species taxa (*S. tenella*, *S. arieticanis*, *S. gigantea*, *S. medusiformis*, and the *S. mihoensis*-like organism), we conducted comparative analyses using the *18S rDNA, 28S rDNA*, and *cox1* loci—the only genetic markers available for all included sheep-infecting *Sarcocystis* species in GenBank (Table 2). At the *18S rDNA* locus, the new species showed the closest genetic affinity to *S. gigantea* (relative genetic distance = 0.0574), followed by *S. medusiformis* (0.0584). Similarly, for the *28S rDNA* locus, the smallest distances were observed with *S. medusiformis* (0.0560) and then *S. gigantea* (0.0610). At the *cox1* locus, the lowest distances were also found with *S. gigantea* (0.2922) and *S. medusiformis* (0.2945).

**Table 2:** Genetic distances between *Sarcocystis chenggongensis* n. sp. and other *Sarcocystis* species infecting sheep

*Sarcosystis* species	*18S rDNA*		*28S rDNA*		*cox1*	
	*S. chenggongensis* n. sp.		*S. chenggongensis* n. sp		*S. chenggongensis* n. sp.	
	Relative distance (mean)	Absolute distance	Relative distance (mean)	Absolute distance	Relative distance	Absolute distance
*S. tenella*	0.0577–0.0594 (0.0585)	162–165	0.0895–0.0908 (0.0903)	286–289	0.3493	288
*S. arieticanis*	0.0611–0.0622 (0.0616)	164–166	0.0852–0.0864 (0.0860)	285–290	0.3637	297
*S. gigantea*	0.0570–0.0581 (0.0574)	160–162	0.0602–0.0617 (0.0610)	225–232	0.2922	250
*S. medusiformis*	0.0580–0.0590 (0.0584)	164–166	0.0556–0.0571 (0.0560)	221–228	0.2945	251
*S. mihoensis*-like	0.0713–0.0730 (0.0721)	206–208	0.1182–0.1210 (0.1190)	320–324	0.5025	373

Genetic distance analysis was conducted by pairwise nucleotide comparisons of homologous gene regions using alignments of 18S rDNA (1989 nt; references: *S. tenella* MK420019, *S. arieticanis* PQ538540, *S. gigantea* MK420020, *S. medusiformis* MK420021, *S. mihoensis*-like MK420022), 28S rDNA (1947 nt; references: *S. tenella* MH413038, *S. arieticanis* MF039327, *S. gigantea* U85706, *S. medusiformis* PV460246, *S. mihoensis*-like MK420028), and *cox*1 (1036 nt; references: *S. tenella* MF039322, *S. arieticanis* MK419975, *S. gigantea* MK420012, *S. medusiformis* MK420014, *S. mihoensis*-like MK420016).

### Phylogenetic analysis

**Figure 2:**
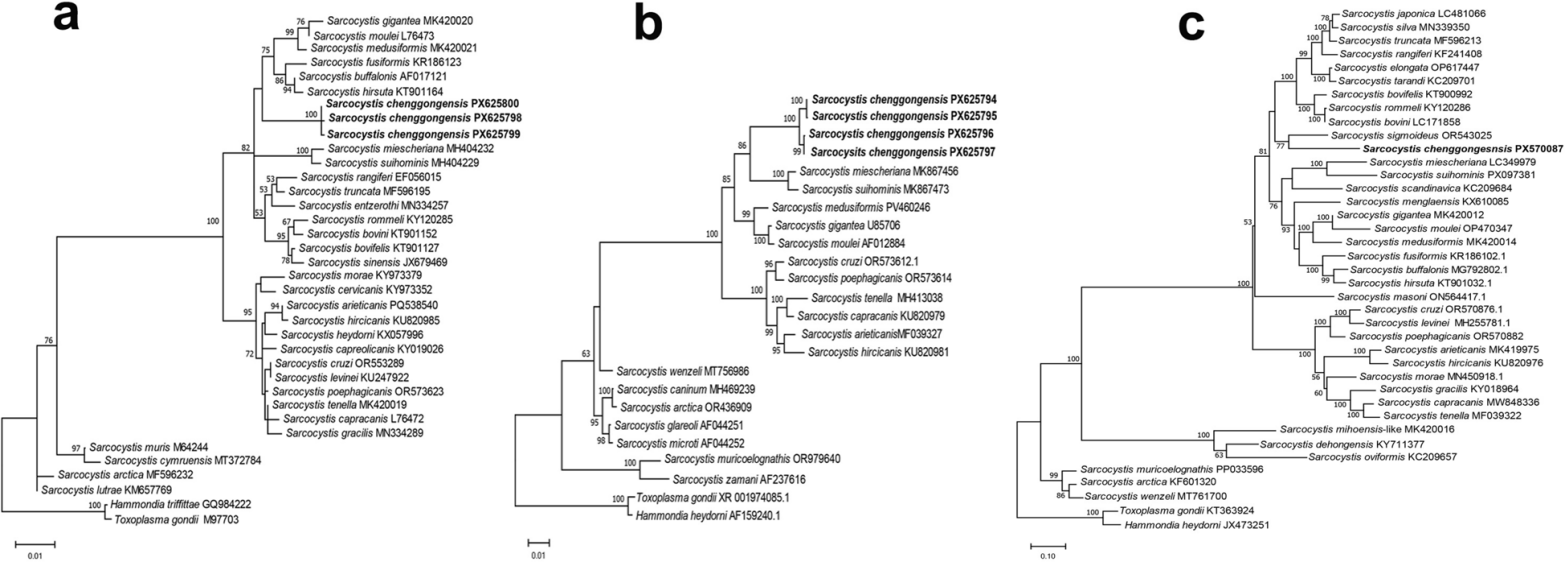
Phylogenetic relationships of selected *Sarcocystis* species based on *18S rDNA* (**a**), *28S rDNA* (**b**), and *cox1* (**c**) sequences. ML trees were reconstructed using the Hasegawa–Kishino–Yano model for *18S* and *28S rDNA*, and the general time reversible model for *cox1*. Numbers at nodes indicate bootstrap support values from 1000 replicates; values below 50% are not shown. *Toxoplasma gondii* and *Hammondia* spp. were designated as outgroups. Newly generated sequences of *S. chengongensis* n. sp.—*18S rDNA* (PX625798–PX625800), *28S rDNA* (PX625794–PX625797), and *cox1* (PX570087)—are highlighted in bold. All three gene trees exhibited congruent topologies, consistently placing *S. chengongensis* within a clade predominantly composed of ruminant-infecting Sarcocystis species that utilize felids as definitive hosts

Phylogenetic trees reconstructed from *18S rDNA* (Fig. 2a), *28S rDNA* (Fig. 2b), and *cox1* (Fig. 2c) sequences consistently placed the novel *Sarcocystis* species within a distinct clade. This clade is composed mainly of species that infect ruminants and use felids as definitive or potential definitive hosts. In the *18S rDNA* phylogenetic tree, the three isolates of the novel species formed a distinct clade that was positioned basally to the clade containing macrocyst-forming *Sarcocystis* species from ruminants. This macrocyst-forming clade includes *S. gigantea* and *S. medusiformis* from sheep, *S. moulei* from goats, and *S. hirsuta* from cattle, as well as *S. fusiformis* and *S. buffalonis* from water buffalo. The clade comprising the novel species and the macrocyst-forming species formed a sister group to a clade of microcyst-forming Sarcocystis species from ruminants. In the *28S rDNA* phylogenetic tree, the four isolates formed a distinct clade, which clustered with *S. miescheriana* and *S. suihominis* from domestic pigs. This combined clade was, in turn, sister to the group comprising *S. medusiformis*, *S. gigantea*, and *S. moulei*. In the *cox1* phylogenetic tree, the novel species clustered with *S. sigmoideus* from cattle, forming a clade positioned basal to a group of microcyst-forming *Sarcocystis* species found in cervids or bovines. The microcyst-forming clade, in turn, was sister to a clade of macrocyst-forming *Sarcocystis* species that infect ruminants.

On the basis of integrated morphological and molecular data, the sarcocysts isolated from domestic sheep in China represent a previously undescribed species. We propose the name *Sarcocystis chenggongensis* n. sp. for this novel taxon.

### Taxonomic summary of *Sarcocystis chenggongensis* n. sp.

*Diagnosis*: The sarcocysts are macroscopic, reaching a maximum length of 6230 μm and a width of 341 μm. Under LM, the cyst wall was adorned with numerous sloping VPs with lengths ranging from 6.9 to 11.9 μm. TEM further elucidated the morphological details of these VPs. The VPs measured 5.8–9.3 μm in length and 0.8–1.1 μm in width. Scattered microtubules traverse the entire length of each VP but do not penetrate the underlying GSL.

*Type intermediate host*: Domestic sheep *Ovis aries*.

*Type locality*: Chenggong District, Kunming City, China.

*Site of infection*: Muscular tissues.

*Definitive host*: Unknown.

*Etymology*: Latin name of the type locality is used to name the species.

*Molecular characterization*: The *18S rDNA* (PX625798–PX625800), *28S rDNA* (PX625794–PX625797), *ITS-1* (PX632648–PX632651), *cox1* (PX570087), and *rpo*B (PX619186) sequences of the novel species have been deposited in GenBank. Molecular analysis of these loci confirms that *S. chenggongensis* is genetically distinct from all known *Sarcocystis* species infecting sheep and other animals.

*Deposited specimens*: Formalin–fixed tissues containing cysts of *S*. *chenggongensis*, as well as photomicrographs from LM and TEM examination of the sarcocysts, have been deposited at the Zoological Specimen Museum of Yunnan University, Kunming, China (collection number Prot202510).

*ZooBank registration*: To comply with the regulations set out in Article 8.5 of the amended 2012 version of the International Code of Zoological Nomenclature [26], details of the new species have been submitted to ZooBank. The Life Science Identifier (LSID) of the article is urn:lsid:zoobank.org:pub:EBE1E880-B86D-42D7-A322-AB6F767D4D59. The LSID for the new species name *Sarcocystis chenggongensis* is urn:lsid:zoobank.org:act: 714F164C-1AC0-47C9-970E-814FD7CCA34F.

### Remarks

To date, nine *Sarcocystis* species are known to form sarcocysts in sheep (Table 3), categorized by sarcocyst size as either microcyst-forming (*S. tenella*, *S. arieticanis*, *S. gracilis*-like, and *S. microps*) or macrocyst-forming (*S. gigantea*, *S. medusiformis*, *S. cystiformis*, *S. mihoensis*, and *S. mihoensis*-like). The newly described *S. chenggongensis* n. sp. forms macrocysts and is readily distinguished from *S. gigantea* and *S. medusiformis* —which possess striated cyst walls without visible protrusions—and from *S. cystiformis*, which has a smooth cyst wall, by its sloping VPs visible by LM. However, differentiation from *S. mihoensis* and *S. mihoensis*-like based on LM morphology alone is challenging due to their similar VP appearance. Ultrastructural analysis resolves this ambiguity: *S. chenggongensis* exhibits finger-like VPs containing scattered microtubules that extend their full length (conforming to TEM wall type 10f), whereas *S. mihoensis* has broad-based, tapering VPs with bundled microtubules, sometimes giving a mushroom-shaped profile (TEM wall type 39). Although ultrastructural data for *S. mihoensis*-like are unavailable, significant genetic divergence from *S. chenggongensis* was confirmed, with the largest observed genetic distances (*18S rDNA*: 0.0721; *28S rDNA*: 0.1190; *cox1*: 0.5025) among sheep-infecting *Sarcocystis* species (Table 2).

**Table 3:** *Sarcocystis* species in domestic sheep

*Sarcocystis* species	Definitive host	Sarcocysts		Available sequences	Location	References
		Light microscopy study	Transmission electron microscopy study			
*S. tenella*	Dog (*Canis lupus*), coyote (*C. latrans*), and red fox (*Vulpes vulpes*)	Microscopic, fusiform, up to 700 μm long; cyst wall (1–3 μm thick) with palisade-like protrusions	Villous or palisade-like protrusions, type 14	*18S rDNA*, *28S rDNA*, *cox1*, *ITS-1*	Worldwide	[1, 17, 27, 28]
*S. arieticanis*	Dog (*C. lupus*)	Microscopic, fusiform, up to 900 μm long; thin cyst wall (< 1 μm thick) with hair-like protrusions	Irregularly folded, but non-branched, hirsute or bone-like protrusions, type 7b	*18S rDNA*, *28S rDNA*, *cox1*, *ITS-1*	Worldwide	[1, 17, 27–29]
*S. gigantea*	Cat (*Felis catus*)	Macroscopic, oval or pear-shaped, up to 1 cm long. Striated cyst wall (< 2 μm thick) with no visible protrusions	Cauliflower-like villar protrusions, type 21	*18S rDNA*, *28S rDNA*, *cox1*,	Worldwide	[1, 15, 18, 30]
*S. medusiformis*	Cat (*F. catus*)	Macroscopic, fusiform, up to 8 mm long; striated cyst wall (< 2 μm thick) with no visible protrusions	Trapezoidal or snake-like villar protrusions, type 20	*18S rDNA*, *28S rDNA*, *cox*1, *ITS-1*	Europe, Asia, and Africa	[1, 15, 30]
*S. mihoensis*	Dog (*C. lupus*)	Macroscopic, fusiform, up to 2.1 mm long; thick cyst wall (10–12 μm in thickness) with sloping VPs	Figure-like villar protrusions; cross section of villar protrusions mush-room-shaped, type 39	No data	Japan	[1, 11]
*S. mihoensis*-like	Unknown	Macroscopic, fusiform, up to 2 mm long; thick cyst wall (15 μm in thickness) with slanting thorn-like protrusions	No data	*18S rDNA*, *28S rDNA*, *cox1*	Spain	[15]
*S. gracilis*-like	Unknown	Microscopic, up to 800 µm long; thick cyst wall (up to 5 μm) with radial striations	Finger-like villar protrusions, VPs tightly packed, upright, type 10e	No data	Italy	[14]
*S. microps*	Dog (*C. lupus*)	Microscopic, fusiform, up to 310 µm long; thin cyst wall (1.3 µm thick)	T-shaped villar protrusions	No data	China	[12]
*S. cystiformi*s	Dog (*C. lupus*)	Macroscopic, sac-like, diameter 3.0–3.5 mm; thick cyst wall (up to 5.5 µm thick)	Cyst wall smooth, no projections	No data	China	[13]
*S. chenggongensis* n. sp.	Unknown	Macroscopic, fusiform, up to 6 mm long; thick cyst wall (up to 12 μm in thickness) with sloping VPs	Sloping VPs, type 10f	*18S rDNA*, *28S rDNA*, *cox1*, *ITS-1*, *rpoB*	China	This study

## Discussion

The ultrastructure of the sarcocyst wall is a traditional and critical taxonomic feature for differentiating *Sarcocystis* species within the same host. A standardized classification system established by Dubey et al. (2016) defines 42 distinct wall types on the basis of these ultrastructural features [1]. Among the known *Sarcocystis* species in sheep, six have well-defined wall types: *S. tenella* (type 14), *S. arieticanis* (type 7b), *S. gigantea* (type 21), *S. medusiformis* (type 20), *S. mihoensis* (type 39), and *S. gracilis*-like (type 10e). In contrast, the wall types for *S. mihoensis*-like, *S. microps*, and *S. cystiformis* remain undetermined due to either a lack of sufficient TEM data (*S. mihoensis*-like) or inconclusive original descriptions with poor-quality micrographs (*S. microps*, *S. cystiformis*). The newly identified *S. chenggongensis* n. sp. is characterized by wall type 10f, defined by long, sloping VPs. This clearly distinguishes it from *S. gracilis*-like (type 10e), which possesses tightly packed, nearly upright VPs [14]. Notably, the original *S. gracilis*, described by Giannetto et al. (2005) in roe deer (*Capreolus capreolus*), forms macroscopic sarcocysts (0.5–2 mm long) with finger-like protrusions (2.5–3 μm long) containing fibrillar elements [31].

Molecular characterization is indispensable for the accurate classification of *Sarcocystis* species due to the limitations of morphological identification. These limitations stem from several sources: (1) morphologically similar sarcocysts frequently occur in different host species (e.g., *S. tenella*, *S. arieticanis*, and *S. gigantea* in sheep closely resemble *S. capracanis*, *S. hircicanis*, and *S. moulei* in goats, respectively) [1, 17, 32]; (2) cyst morphology is developmentally plastic, with changes in size and villar projection structure over time [33–35]; and (3) the observed ultrastructure of the cyst wall can vary substantially with the sectioning plane [11, 36]. These challenges have resulted in considerable taxonomic confusion, particularly among ruminant-infecting *Sarcocystis* species [37–40], underscoring the need for molecular markers for reliable differentiation. While the nuclear *18S rDNA* gene is often too conserved to distinguish closely related ruminant-infecting species, the mitochondrial *cox1* gene provides superior phylogenetic resolution due to its higher divergence [22].

In this study, we sequenced five genetic loci from *S. chenggongensis* n. sp. and compared them with homologous sequences from other *Sarcocystis* spp., with a particular focus on sheep-infecting species. Notably, none of the sequenced loci showed the highest similarity to known sheep-associated species. The *18S rDNA* sequences exhibited the greatest similarity (97.6–98.0%) to *S. buffalonis* from water buffalo, while the *cox1* sequences were most closely related (81.2–81.8%) to *S. japonica* from sika deer. Both conserved and variable genetic markers clearly distinguished *S. chenggongensis* from all other documented *Sarcocystis* species.

To further validate its taxonomic status, we analyzed the genetic distances between *S. chenggongensis* n. sp. and other sheep-associated *Sarcocystis* species. Despite its light-microscopic resemblance to *S. mihoensis*-like cysts, genetic distance analysis revealed that the novel species was most closely related to *S. gigantea* and *S. medusiformis*. In fact, at all loci examined, *S. chenggongensis* n. sp. exhibited the greatest genetic divergence from the *S. mihoensis*-like organism among the five sheep-infecting species with available sequence data (*S. tenella*, *S. arieticanis*, *S. gigantea*, *S. medusiformis*, and the *S. mihoensis*-like organism) (Table 2). The combined morphological and molecular evidence robustly supports the recognition of *S. chenggongensis* as a distinct species within the *Sarcocystis* genus.

Phylogenetic analysis is a well-established tool for identifying the potential definitive hosts of *Sarcocystis* spp., as it reflects their co-evolutionary relationships [41]. Specifically, ruminant-infecting *Sarcocystis* species consistently form distinct phylogenetic clades corresponding to whether their definitive hosts are canids or felids [42, 43]. To date, only canids and felids have been confirmed as definitive hosts for *Sarcocystis* species with known life cycles in domestic sheep. Among these, feline-associated species that form macrocysts, such as *S. gigantea* and *S. medusiformis*, typically exhibit lower prevalence than canine-associated, microcyst-forming species (e.g., *S. tenella* and *S. arieticanis*) [1, 44]. This disparity is likely due to the relatively lower infectivity of macrocysts in cats [35]. The phylogenetic placement of *S. chenggongensis* n. sp. within a clade of macrocyst-forming, feline-associated species, coupled with its low prevalence (7.2%) in the local sheep population compared with the highly prevalent (91.9%) microcyst-forming, canine-associated species [17], suggests the domestic cat is its probable definitive host. This proposed life cycle is consistent with the local practice of feeding raw mutton to cats.

## Conclusions

While nine *Sarcocystis* species have been reported in domestic sheep, only four—*S. tenella*, *S. arieticanis*, *S. gigantea*, and *S. medusiformis*—are well documented. The remaining species are either rare or inadequately characterized. Macroscopic sarcocysts with elongated, villar protrusions are particularly rare, having been documented on only three occasions: *S. mihoensis* in Japan, a *S. mihoensis*-like species in Spain, and now *S. chenggongensis* in China. This suggests a wide but sporadic geographical distribution for this morphotype. Taxonomic classification of sheep-infecting *Sarcocystis* remains challenging due to inconsistent nomenclature and a lack of molecular and life cycle data. For instance, while the dog has been experimentally confirmed as the definitive host for *S. mihoensis*, molecular verification is still pending. In this study, *S. chenggongensis* was unequivocally established as a new species through ultrastructural and molecular analyses, with preliminary evidence pointing to the domestic cat as its potential definitive host. Future research should focus on resolving the evolutionary relationships and life cycles of these morphologically similar species to advance the taxonomy and epidemiology of these parasites.

## Data Availability

Data supporting the main conclusions of this study are included in the manuscript. Nucleotide sequences of the *18S rDNA* (PX625798–PX625800), *28S rDNA* (PX625794–PX625797), *ITS-1* (PX632648–PX632651), *cox1* (PX570087), and *rpoB* (PX619186) of the new species have been deposited in GenBank.

## Abbreviations

LM: Light microscopy
TEM: Transmission electron microscopy
H&E: Hematoxylin and eosin
VPs: Villus protrusions
GSL: Ground substance layer
MT: Microtubules
HC: Host cell
18S rDNA: Small subunit ribosomal RNA gene
28S rDNA: Large subunit ribosomal RNA gene
ITS-1: Internal transcribed spacer 1
*cox*1: Cytochrome oxidase subunit 1
*rpoB*: RNA polymerase beta subunit gene
ML: Maximum likelihood
